# LGBTQIA+ OLDER ADULTS’ NEEDS, CONCERNS, AND EXPERIENCES IN HEALTH CARE: INCLUSIVITY, CAREGIVING, AND VISIBILITY

**DOI:** 10.1093/geroni/igae098.0795

**Published:** 2024-12-31

**Authors:** Korijna Valenti, Sara Bybee

**Affiliations:** Chair; Discussant

## Abstract

LGBTQIA+ aging research requires in-depth examination of experiences around discrimination, inclusivity, and communication in healthcare to better understand the distinct needs of diverse subgroups, including people living in long-term care (LTC) facilities, living with neurodegenerative illnesses, and caregivers. LGBTQIA+ older adults not only have diverse life experiences but also specific needs regarding advocacy, policy, and healthcare support and resources. This symposium includes findings from four distinct areas of study. Valenti and colleagues examined perspectives of 20 palliative-cared trained clinicians to better understand their experiences with LGBTQI+ patients living with serious illness and their chosen care partners, exploring issues around communication, collection of SOGI data, and inclusivity. In a study with LTC administrators in Minnesota, Wright and colleagues examined issues around identifying LGBTQIA+ LTC residents, discrimination of residents (including residents living with cognitive impairment), and barriers around LGBTQIA+ inclusion in research. Stein and colleagues examined issues around discrimination in healthcare for LGBTQIA+ patients through Project Respect, a cross-sectional mixed methods study of 290 patients and partners which found high levels of disrespect and inadequate care. Flatt and colleagues provide insights from an analysis of two qualitative studies of AD/PD caregivers with a specific focus on identifying barriers to support, inclusion in research, and issues around personal and social needs. Presentations in this symposium illustrate how discrimination, inclusivity, and personal and social support inform the needs of LGBTQIA+ older adults living with serious illness, in LTC, and as caregivers as well as providing implications for future aging research and interventions in practice.

